# Age at lung cancer diagnosis in females versus males who never smoke by race and ethnicity

**DOI:** 10.1038/s41416-024-02592-z

**Published:** 2024-02-22

**Authors:** Batel Blechter, Jason Y. Y. Wong, Li-Hsin Chien, Kouya Shiraishi, Xiao-Ou Shu, Qiuyin Cai, Wei Zheng, Bu-Tian Ji, Wei Hu, Mohammad L. Rahman, Hsin-Fang Jiang, Fang-Yu Tsai, Wen-Yi Huang, Yu-Tang Gao, Xijing Han, Mark D. Steinwandel, Gong Yang, Yihe G. Daida, Su-Ying Liang, Scarlett L. Gomez, Mindy C. DeRouen, W. Ryan Diver, Ananya G. Reddy, Alpa V. Patel, Loïc Le Marchand, Christopher Haiman, Takashi Kohno, Iona Cheng, I-Shou Chang, Chao Agnes Hsiung, Nathaniel Rothman, Qing Lan

**Affiliations:** 1 Division of Cancer Epidemiology and Genetics, National Cancer Institute, Rockville, MD USA; 2 Institute of Population Health Sciences, National Health Research Institutes, Zhunan, Taiwan; 3 Department of Applied Mathematics, Chung-Yuan Christian University, Chung-Li, Taiwan; 4 Division of Genome Biology, National Cancer Center Research Institute, Tokyo, Japan; 5 Department of Clinical Genomics, National Cancer Center Research Institute, Tokyo, Japan; 6 Division of Epidemiology, Department of Medicine, Vanderbilt University Medical Center and Vanderbilt-Ingram Cancer Center, Nashville, TN USA; 7 National Institute of Cancer Research, National Health Research Institutes, Zhunan, Taiwan; 8 Department of Epidemiology, Shanghai Cancer Institute, Shanghai, China; 9 Vanderbilt Institute for Clinical and Translational Research, Vanderbilt University Medical Center, Nashville, TN USA; 10 Center for Integrated Health Care Research, Kaiser Permanente Hawai’i, Honolulu, HI USA; 11 Palo Alto Medical Foundation Research Institute, Sutter Health, Palo Alto, CA USA; 12 Greater Bay Area Cancer Registry, University of California, San Francisco, CA USA; 13 Department of Epidemiology & Biostatistics, University of California, San Francisco, CA USA; 14 Helen Diller Family Comprehensive Cancer Center, University of California, San Francisco, CA USA; 15 Department of Population Science, American Cancer Society, Kennesaw, GA USA; 16 University of Hawaii Cancer Center, Honolulu, HI USA

**Keywords:** Lung cancer, Lung cancer

## Abstract

**Background:**

We characterized age at diagnosis and estimated sex differences for lung cancer and its histological subtypes among individuals who never smoke.

**Methods:**

We analyzed the distribution of age at lung cancer diagnosis in 33,793 individuals across 8 cohort studies and two national registries from East Asia, the United States (US) and the United Kingdom (UK). Student’s t-tests were used to assess the study population differences (Δ years) in age at diagnosis comparing females and males who never smoke across subgroups defined by race/ethnicity, geographic location, and histological subtypes.

**Results:**

We found that among Chinese individuals diagnosed with lung cancer who never smoke, females were diagnosed with lung cancer younger than males in the Taiwan Cancer Registry (*n* = 29,832) (Δ years = −2.2 (95% confidence interval (CI):−2.5, −1.9), in Shanghai (*n* = 1049) (Δ years = −1.6 (95% CI:-2.9, −0.3), and in Sutter Health and Kaiser Permanente Hawaiʽi in the US (*n* = 82) (Δ years = −11.3 (95% CI: −17.7, −4.9). While there was a suggestion of similar patterns in African American and non-Hispanic White individuals. the estimated differences were not consistent across studies and were not statistically significant.

**Conclusions:**

We found evidence of sex differences for age at lung cancer diagnosis among individuals who never smoke.

## Introduction

Lung cancer is a significant global health burden with an estimated 2.2 million new cases and 1.8 million deaths worldwide in 2020 [1]. While active smoking is the most prominent risk factor for lung cancer, nearly 25% of lung cancer cases diagnosed worldwide are among people who never smoke, with the proportion varying geographically [2]. It is estimated that 10% of lung cancer cases in the United States (US) and as high as 50% in Asia are diagnosed among patients who never smoke, with higher proportions among female populations [3]. Among lung cancer patients who never smoke, lung adenocarcinoma (LUAD), which originates in the mucous-producing epithelial lining of the lung, is the most commonly diagnosed histological subtype [2, 4], with a higher proportion among females compared to males [4, 5].

Lung cancer is a highly fatal disease, with an overall 5-year survival rate of 22.9% in the US from 2012 to 2018, which ranges from 61.2% for early (localized) stage to as low as 7% for distant stage (metastasized) [6]. Age at diagnosis is an important prognostic factor that influences clinical treatment decisions and survival of lung cancer patients [7–11], as those with younger age at diagnosis often present with earlier tumor stages and benefit from early targeted treatment [12, 13]. According to the Surveillance, Epidemiology, and End Results Program (SEER), the median age at lung cancer diagnosis in the US is 71 years. However, evidence from cancer registries, patient medical records, and case-series analyses suggest differences in age at lung cancer diagnosis between subpopulations defined by smoking status, race and ethnicity, and geographic location [14–24]. Notably, some studies have reported differences with females more often diagnosed [25, 26] at younger ages [27–29], with LUAD [28, 30, 31], and with earlier tumor stage at diagnosis, compared to males [25, 26]. However, the existence of sex differences in age at lung cancer diagnosis among individuals who never smoke, which may contribute to lung cancer-related disparities between females and males [25, 26, 28–31], are unclear.

Given the importance of age at diagnosis for disease prognosis, clinical treatment decisions, and survival of lung cancer patients [6–10], we conducted an international study to characterize age at diagnosis for lung cancer patients who never smoke, as well as examined sex differences by histological subtypes (i.e., LUAD and squamous cell carcinoma [SCC]) and race and ethnicity.

## Methods

### Study Design

The lung cancer case ascertainment procedures and criteria, as well as histological subtype confirmation for each participating study have been described in detail elsewhere [24, 32–51]. We received data on age at lung cancer diagnosis among patients who never smoke by self-reported sex, race and ethnicity, as well as histology and SEER tumor stage (when available) from 8 cohort studies and two population-based cancer registries. Individual-level data were provided for 1,571 lung cancer patients who never smoke from the Shanghai Women’s Health Study (SWHS; *n* = 760) [52], the Shanghai Men’s Health Study (SMHS; *n* = 289) [40], the Prostate, Lung, Colorectal, and Ovarian Cancer (PLCO) Screening Trial (*n* = 39) [41], and the UK Biobank (*n* = 483) [42, 43]. Summary statistics (i.e., mean, standard deviation (SD), min, max, median, 25th and 75th percentile) were provided for 32,222 lung cancer cases who never smoke from the Taiwan Cancer Registry (TCR) [32, 33] population study (*n* = 29,832), the Multiethnic Cohort Study (MEC; *n* = 578) [34], the Cancer Prevention Study-II Nutrition Cohort and Cancer Prevention Study-3 (CPSII, *n* = 365; CPS-3, *n* = 64) [35, 36], Sutter Health of Northern California and Kaiser Permanente Hawaiʽi (STKP; *n* = 515) [24, 37], the Southern Community Cohort Study (SCCS; *n* = 138) [38, 39], and the National Cancer Center Japan (NCC; *n* = 730) [53]. In total, we received data for 33,793 lung cancer cases.

Each participating study obtained its own institutional review board approval. Sutter Health of Northern California and Kaiser Permanente Hawaiʽi received waiver of consent, and consent for participants in MEC was received with receipt of baseline questionnaire. All remaining participants provided written or electronic informed consent.

### Statistical analyses

In each of the participating studies, we characterized the distribution of age at diagnosis of lung cancer and its histological subtypes (i.e., LUAD and SCC) among individuals who never smoke by self-reported biological sex (i.e., male, female), race and ethnicity (i.e., Chinese and Japanese individuals living in Asia, Chinese and Japanese individuals living in the US, non-Hispanic White and African American individuals living in the US, and White individuals living in the UK), and SEER tumor stage (i.e., local, regional, distant) for studies with available data. Within each participating study, we estimated differences in average age at diagnosis of overall lung cancer, LUAD, and SCC between females and males who never smoke and tested the differences using Student’s t-tests. The differences in average age at diagnosis (Δ years) between females and males were then meta-analyzed across populations of similar racial and ethnic subgroup using random effects inverse variance method. All analyses were performed using the R statistical software (version 4.2.2).

## Results

Information on each participating study, including study design, years of enrollment, geographical region, type of data provided, sample size and criteria for age at enrollment is shown in Table 1. Study specific and combined estimates for the difference in age at diagnosis for lung cancer comparing East Asian females and males who never smoke living in Taiwan, China, and US, overall and by histological subtype are presented in Table 2. We observed that among individuals who never smoke in TCR, a population-based registry and the largest study in our analyses, and in the SWHS and SMHS, large population-based prospective cohort studies of Chinese individuals, females were diagnosed younger than males by 2.2 years (Δ years = −2.2 (95% confidence interval (CI): −2.5, −1.88; P_difference_ = 2.19 × 10^−^^40^) and 1.6 years (Δ years = −1.6 (95% CI: −2.9, −0.3; P_difference_ = 0.02), respectively. We observed similar patterns in the Chinese populations living in East Asia for LUAD and SCC. Further, we found that Chinese females who never smoke living in the US from STKP were also diagnosed with overall lung cancer younger than males (Δ years = −11.3 (95% CI: −17.7, −4.9; P_difference_ 5.38 x 10^−4^). Conversely, we observed that among Japanese individuals who never smoke living in Japan from the NCC, the age at LUAD diagnosis was older for females compared to males (Δ years = 3.3 (95% CI: 1.0, 5.6; P_difference_ = 5.00 × 10^-3^). This pattern was further supported in Japanese individuals who never smoke living in the US from MEC, where females had a suggestive older age at LUAD diagnosis compared to males (Δ years = 2.9 (95% CI: −0.2, 6.0; P_difference_ = 0.07) (Supplementary Table 1).

**Table 1:** Characteristics of participating studies

Study	Country or Region	Study Design	Study start/recruitment year(s)	Age at recruitment, years	Data level	N^a^
Taiwan Cancer Registry Population Study [32, 33]	Taiwan	Population Registry	1979^b^	NA^c^	Summary	29,832
Shanghai Women’s Health Study/Shanghai Men’s Health Study [40, 52]	Mainland China	Prospective Cohort	1996−2000; 2002	40–70; 40–74^d^	Individual	1049
Multiethnic Cohort Study [51]	California, Hawai'i, USA	Prospective Cohort	1993–1996	45–75	Summary	578
National Cancer Center Japan [53]	Japan	Case series^e^	1962	NA^c^	Summary	730
Sutter Health of Northern California and Kaiser Permanente Hawai’i [24, 37]	Northern California, USA	Prospective Cohort with Electronic Medical Records linked to state cancer registries	2000–2013	NA^c^	Summary	515
UK Biobank (European ancestry only) [42, 43, 48, 49]	United Kingdom	Prospective Cohort	2006–2010	40–69	Individual	483
Cancer Prevention Study-II Nutrition Cohort [35]	USA	Prospective Cohort	1992	40–92	Summary	365
Southern Community Cohort Study [38]	USA	Electronic Medical Record-based cohort^f^	2002–2009	40–79	Summary	138
Cancer Prevention Study-3 [36]	USA	Prospective Cohort	2006–2013	30–65	Summary	64
Prostate, Lung, Colorectal, and Ovarian Cancer (PLCO) Screening Trial (European ancestry only) [41, 47]	USA	Prospective Cohort, Screening Trial	1993–2001	55–74	Individual	39

^a^Number of lung cancer cases included in analyses

^b^Data used for analyses was from 2011 to 2016, when smoking status became available

^c^Studies linked to registries with no inclusion criteria based on age

^d^Analyses excluded patients age > 70 in the Shanghai Men’s Health Study for comparability with the Shanghai Women’s Health Study

^e^Data provided was from a case series, which is under representative of the population as age at diagnosis may have differentially been influenced by referral patterns.

^f^Exposure data (including smoking) were prospectively collected via electronic medical records

**Table 2:** Differences in age at lung cancer diagnosis among Chinese females and males who never smoke

Study/ Lung cancer histological subtype	Females			Males			Differences in age at diagnosis between females and males	
	*n*	Age at diagnosis, years, mean (SD)		*n*	Age at diagnosis, years, mean (SD)		Estimate (95% CI) ^b^	*P*-value
Taiwan Cancer Registry Population Study								
Overall^a^	21343	65.6 (12.7)		8489	67.8 (13.2)		−2.2 (−2.5, −1.9)	2.19 × 10^-40^
LUAD	18735	65.2 (12.5)		6086	66.1 (12.9)		−0.9 (−1.3, −0.5)	1.92 × 10^-6^
SCC	778	67.2 (12.6)		1013	72.4 (11.6)		−5.2 (−6.3, −4.1)	7.21 × 10^-19^
Shanghai Women’s and Men’s Health Study								
Overall^a^	760	67.1 (9.1)		289	68.7 (9.8)		−1.6 (−2.9, −0.3)	0.02
LUAD	409	65.2 (8.4)		109	66.1 (8.9)		−0.9 (−2.8, 1.0)	0.34
SCC	20	63.0 (9.2)		41	67.2 (9.4)		−4.2 (−9.3, 0.9)	0.10
Sutter Health of Northern California and Kaiser Permanente Hawai’i								
Overall^a^	59	59.2 (16.8)		23	70.5 (11.3)		−11.3 (−17.7, −4.9)	5.38 x 10^−4^
Random effects meta-analysis of differences in age at diagnosis between women and men								
			Chinese patients living in Asia (Taiwan, SWHS/SMHS)					
			Estimate (95% CI) ^c^			*P*-value		
Overall			−2.2 (−2.5, −1.9)			1.20 × 10^-40^		
LUAD			−0.9 (−1.3, −0.5)			1.37 × 10^-6^		
SCC			−5.1 (−6.2, −4.0)			1.68 × 10^-19^		

*SD* Standard deviation, *CI* Confidence interval, *LUAD* Lung adenocarcinoma, *SCC* Squamous cell carcinoma

^a^Overall lung cancer includes LUAD, SCC, as well as other histological subtypes

^b^Estimated difference between mean age at diagnosis and 95% confidence intervals were derived using Student’s t-test

^c^Estimates and 95% confidence intervals for meta-analyses were derived using the inverse variance method

Study specific and combined estimates for the difference in age at diagnosis for lung cancer comparing non-Hispanic White females and males living in the US and White females and males living in the UK overall and by histological subtype are presented in Table 3. Similar to the Chinese population, we observed that non-Hispanic White females living in the US from CPSII were diagnosed with overall lung cancer younger than males (Δ years = −2.0 (95% CI: −3.7, −0.3; P_difference_ = 0.02). While less pronounced, the meta-analysis across the 7 studies with non-Hispanic White and White individuals who never smoke living in the US and UK suggested that females had younger age at diagnosis compared to males for overall lung cancer (Δ years_combined_ = −0.8 (95% CI: −1.9, 0.4; P_meta-analysis_ = 0.18) and LUAD (Δ years_combined_ = −1.1 (95% CI: −2.4, 0.1; P_meta-analysis_ = 0.08); however, the findings were not consistent across studies and were not statistically significant. We further found that among African American individuals who never smoke in the two studies with available data (SCCS and MEC), females had younger age at lung cancer diagnosis compared to males (Δ years_combined_ = −1.0 (95% CI: −4.0, 1.9; P_meta-analysis_ = 0.49); however, the sample size was limited and the findings were not statistically significant (Supplementary Table 2).

**Table 3:** Differences in age at lung cancer diagnosis among non-Hispanic White and White European females and males who never smoke

Study/ Lung cancer histological subtype	Females			Males		Differences in age at diagnosis between females and males	
	*n*		Age at diagnosis, years, mean (SD)	*n*	Age at diagnosis, years, mean (SD)	Estimate (95% CI)^b^	*P*-value
Sutter Health of Northern California and Kaiser Permanente Hawai’i							
Overall^a^	306		64.4 (14.0)	127	64.3 (14.7)	0.1 (−2.9, 3.1)	0.95
Southern Community Cohort Study							
Overall^a^	28		70.3 (8.7)	9	74.3 (11.2)	−4.0 (-13.0, 5.0)	0.35
LUAD	14		68.6 (7.5)	1	NA	NA	NA
SCC	-		-	2	73.0 (19.8)	NA	NA
Cancer Prevention Study-II Nutrition Cohort							
Overall^a^	237		75.0 (7.7)	128	77.0 (7.6)	−2.0 (−3.7, −0.3)	0.02
LUAD	149		76.1 (7.1)	77	76.5 (7.5)	−0.4 (−2.4, 1.6)	0.70
SCC	7		73.0 (5.6)	5	82.2 (4.2)	−9.2 (−15.5, −2.9)	0.01
Cancer Prevention Study-3							
Overall^a^	55		55.7 (7.3)	9	57.2 (11.7)	−1.53 (−10.6, 7.6)	0.72
LUAD	38		56.2 (6.5)	6	54.7 (13.9)	1.5 (−13.1, 16.1)	0.80
Multiethnic Cohort							
Overall^a^	101		75.1 (8.9)	49	75.2 (10.5)	−0.1 (−3.6, 3.4)	0.95
LUAD	56		74.8 (8.2)	21	76.7 (6.9)	−1.9 (−5.7, 1.9)	0.31
SCC	4		68.3 (9.0)	3	68.0 (11.3)	0.3 (−22.2, 22.8)	0.97
UK Biobank							
Overall^a^	307		68.0 (6.8)	176	68.0 (7.1)	0.01 (−1.3, 1.3)	0.99
LUAD	151		67.1 (6.5)	75	68.7 (6.8)	−1.6 (−3.5, 0.3)	0.09
SCC	21		69.3 (5.6)	14	68.2 (5.2)	1.1 (−2.7, 4.9)	0.56
PLCO							
Overall^a^	24		70.7 (5.6)	15	71.9 (5.6)	−1.2 (−5.0, 2.6)	0.52
LUAD	21		71.2 (5.6)	5	72.1 (4.9)	−0.9 (−6.9, 5.1)	0.73
SCC	-		-	3	69.9 (5.5)	NA	NA
Random effects meta-analysis of differences in age at diagnosis between women and men							
		Estimate (95% CI)^c^			*P*-value		
Overall		−0.8 (−1.9, 0.4)			0.18		
LUAD		−1.1 (−2.4, 0.1)			0.08		
SCC		−3.2 (−11.5, 5.1)			0.45		

*SD* Standard deviation, *CI* Confidence interval, *LUAD* Lung adenocarcinoma, *SCC* Squamous cell carcinoma, *PLCO* Prostate, Lung, Colorectal, and Ovarian Cancer Screening Trial

^a^Overall lung cancer includes LUAD, SCC, as well as other histological subtypes

^b^Estimated difference between mean age at diagnosis and 95% confidence intervals were derived using Student’s t-test

^c^Estimates and 95% confidence intervals for meta-analyses were derived using the inverse variance method

In additional analyses examining differences in age at lung cancer diagnosis across tumor stages (local, regional, distant) for subjects in TCR, we found among individuals who never smoke, females were diagnosed with lung cancer younger than males for local (Δ years = −1.8 (95% CI: −2.4, −1.2; P_difference_ = 1.68 × 10^−^^8^), regional (Δ years = −3.1 (95% CI: −4.1, −2.2; P_difference_ = 2.18 × 10^−^^10^), and distant tumor stage (Δ years = −1.9 (95% CI: −2.31, −1.49; P_difference_ = 1.14 × 10^-20^). Similar patterns were found for LUAD and SCC in analyses stratified by tumor stage (Supplementary Table 3).

## Discussion

We conducted a large-scale study to characterize age at diagnosis for lung cancer and its histological subtypes, comparing females versus males by race and ethnicity. We analyzed summary and individual-level data from 33,793 lung cancer cases who never smoke in 10 studies from Taiwan, China, Japan, US, and UK. Overall, we found that among Chinese individuals living in East Asia and in the US, females were diagnosed with lung cancer younger than males. While there was a suggestion of similar patterns in African American and non-Hispanic White individuals living in the US and White individuals living in the UK, the estimated differences were not consistent across studies and were not statistically significant. These findings were consistent across histological subtypes, as well as tumor stages. An exception to this pattern was found among Japanese individuals who never smoke living in Japan and in the US, where females were diagnosed with lung cancer at a later age compared to males.

The demographic makeup of lung cancer has substantially shifted over the last few decades, with the proportion of never-smoking lung cancer increasing due to the decline in smoking prevalence, and sex differences in the epidemiology, pathogenesis and outcomes of the disease have become apparent [29, 54]. Factors that may contribute to sex differences in the characteristics of lung cancer, such as age at diagnosis, may include screening, as well as endogenous and exogenous exposures. A study in the US on gender differences in cancer screening behaviors have showed that women are more likely than men to have more frequent contact with health care providers and seek cancer screening [55], which may on average lead to an earlier diagnosis. However, given that our study focused on individuals who never smoke, a population for which there are no established lung cancer screening guidelines, we suspect that screening behavior had limited impact on the observed differences. Further, we found that females had consistent younger age at lung cancer diagnoses across different tumor stages, suggesting that additional factors may impact the observed sex differences.

Endogenous factors that potentially contribute to sex differences in age at diagnosis among lung cancer in patients who never smoke include the distribution of lung cancer histological subtypes, tumor mutations and genetic susceptibility [44, 45, 56–59]. For example, females are at an increased risk compared to males of developing lung cancer with a driver mutation, such as the epidermal growth factor receptor (EGFR) [60–62]. However, studies have found that lung cancer patients with an EGFR mutation are more likely to have an older age at diagnosis [63], and therefore may not be the reason for our findings. Additionally, estrogen may also lead to sex differences in lung cancer. While research on this topic is ongoing, estrogen receptors have been shown to be overexpressed in many lung cancers [61, 64] and studies have linked the use of hormone replacement therapy [65] and reproductive factors to the risk of lung cancer in females [66–68].

Exogenous factors, such as environmental and occupational exposure profiles may also impact sex differences in lung cancer diagnosis. For example, lung cancer risk factors among East Asian individuals who never smoke include indoor solid fuel combustion, fumes from cooking oil, outdoor air pollution, diesel exhaust, workplace exposures, and secondhand smoke [2, 4, 69–74]. Notably, East Asian women are more likely to be exposed to indoor air pollution from fuel and cooking oil early in life [75], as well as to spousal secondhand smoke. Conversely, male individuals in East Asia are more likely to be exposed to occupational sources of air pollution, such as secondhand smoke in the work environment. Despite potential differences between East Asian individuals living in Asia and the US, we found similar patterns of age at diagnoses, which could suggest similar exposures or genetic predisposition.

Our study had notable strengths. We compiled a large pool of ethnically and geographically diverse data of lung cancer cases who never smoke, which allowed for comparison of age at lung cancer diagnosis by sex, within subpopulations defined by race and ethnicity, histological subtype, and tumor stage. Importantly, we obtained data from the TCR, one of the largest sources of lung cancer data for patients who never smoke in the world, which included information on tumor stage. Further, we obtained data from the CPSII, MEC and Sutter Health / Kaiser Permanente- Hawaiʽi, which greatly improved the representation in our study of Chinese and Japanese individuals living in the US.

Our study had some limitations and interpretation of our results should be done with caution. This study was intended primarily as a descriptive analysis as we could not account or adjust for potential sources of confounding or heterogeneity that potentially drive differences in age at diagnosis across the participating studies and their subgroups. Given the heterogeneity among the participating studies, we opted not to calculate a combined weighted average age at diagnosis across studies, as this would not be meaningful. Further, our analyses were primarily conducted in cohort studies, which do not represent the general population. Despite these caveats, a consistently younger age at diagnosis among females versus males was observed across multiple studies, including a large population-based registry, as well as across histological subtypes and tumor stage. Additionally, although some data on African American individuals were obtained from SCCS, MEC, and CPSII, we did not have sufficient case numbers to comprehensively analyze this population and its subgroups. Future studies that include larger African American, as well as Hispanic populations will be needed to accurately characterize differences in age at diagnosis.

In summary, we characterized the average age at diagnosis for lung cancer and its subtypes among patients who never smoke in a comprehensive analysis of 33,793 lung cancer patients from the US, China, Taiwan, Japan and the UK. We found that, among individuals who never smoke, females had a younger age at diagnosis compared to males, which was especially apparent among Chinese individuals living in Taiwan and Mainland China, as well as among Chinese individuals living in the US. Future studies are needed to elucidate the potential reasons for these observed differences.

### Supplementary information

Age at lung cancer diagnosis in females versus males who never smoke by race and ethnicity

## Data Availability

Summary data can be made available upon request.
