# Supplementary material for: Age at lung cancer diagnosis in females versus males who never smoke by race and ethnicity
Source: Br J Cancer. 2024 Feb 22;130(8):1286–94. doi: 10.1038/s41416-024-02592-z (PMC11014844; doi:10.1038/s41416-024-02592-z)
Supplement: Supplementary file 1 — Age at lung cancer diagnosis in females versus males who never smoke by race and ethnicity [file 41416_2024_2592_MOESM1_ESM.docx]

Supplementary Table 1. Differences in age at lung cancer diagnosis among Japanese females and males who never smoke living in Japan and in the United States

| Study/ Lung cancer histological subtype | Females | | Males | | Differences in age at diagnosis between females and males | |
| --- | --- | --- | --- | --- | --- | --- |
|  | n | Age at diagnosis, years, mean (SD) | n | Age at diagnosis, years, mean (SD) | Estimate (95% CI)^b^ | P-value |
| National Cancer Center Japan* | | |  |  |  |  |
| LUAD | 625 | 59.9 (8.1) | 105 | 56.6 (11.4) | 3.3 (1.0, 5.6) | 5.00x10^-3^ |
| Multiethnic Cohort Study (Japanese only) | |  |  |  |  |  |
| Overall^a^ | 225 | 78.4 (9.0) | 80 | 77.1 (9.2) | 1.3 (-1.1, 3.7) | 0.28 |
| LUAD | 129 | 78.5 (8.5) | 49 | 75.6 (9.7) | 2.9 (-0.2, 6.0) | 0.07 |
| SCC | 15 | 80.0 (8.5) | 6 | 70.3 (6.6) | 9.7 (2.1, 17.3) | 0.02 |
| Random effects meta-analysis of differences in age at diagnosis between women and men | | | | | | |
|  | Estimate (95% CI)^c^ | | | P-value | | |
| LUAD | 3.1 (1.3, 5.0) | | | 8.40x10^-4^ | | |

Abbreviations: SD, standard deviation; CI, confidence interval; LUAD, lung adenocarcinoma; SCC, squamous cell carcinoma

^a^ overall lung cancer includes LUAD, SCC, as well as other histological subtypes

^b^ Estimated difference between mean age at diagnosis and 95% confidence intervals were derived using Student’s t-test

^c^ Estimates and 95% confidence intervals for meta-analyses were derived using the inverse variance method

^*^Data provided was from a case series, which is under representative of the population as age at diagnosis may have differentially been influenced by referral patterns.

Supplementary Table 2. Differences in age at lung cancer diagnosis among African American females and males who never smoke

| Study/ Lung cancer histological subtype | Females | | Males | | Differences in age at diagnosis between females and males | |
| --- | --- | --- | --- | --- | --- | --- |
|  | n | Age at diagnosis, years, mean (SD) | n | Age at diagnosis, years, mean (SD) | Estimate (95% CI)^b^ | P-value |
| Multiethnic Cohort Study |  |  |  |  |  |  |
| Overall^a^ | 91 | 75.3 (9.06) | 32 | 77.4 (10.0) | -2.1 (-6.1, 1.9) | 0.30 |
| LUAD | 42 | 74.2 (8.2) | 12 | 79.2 (9.9) | -5.0 (-11.6, 1.6) | 0.13 |
| SCC | 2 | 71.3 (19.8) | 3 | 75.4 (14.4) | -4.1 (-86.2, 78.0) | 0.83 |
| Southern Community Cohort Study |  |  |  |  |  |  |
| Overall^a^ | 67 | 64.3 (10.1) | 34 | 64.1 (10.2) | 0.2 (-4.1, 4.5) | 0.93 |
| LUAD | 36 | 64.6 (9.6) | 9 | 63.0 (11.2) | 1.6 (-7.3, 10.5) | 0.70 |
| SCC | 5 | 57.4 (14.8) | 5 | 66.8 (9.1) | -9.4 (-28.0, 9.2) | 0.27 |
| Random effects meta-analysis of differences in age at diagnosis between women and men | | | | | | |
|  | Estimate (95% CI)^c^ | | | P-value | | |
| Overall | -1.0 (-4.0, 1.9) | | | 0.49 | | |
| LUAD | -2.4 (-8.7, 4.0) | | | 0.46 | | |
| SCC | -9.1 (-27.3,9.0) | | | 0.32 | | |

Abbreviations: SD, standard deviation; CI, confidence interval; LUAD, lung adenocarcinoma; SCC, squamous cell carcinoma; MEC, Multiethnic Cohort Study

^a^ Overall lung cancer includes LUAD, SCC, as well as other histological subtypes

^b^ Estimated difference between mean age at diagnosis and 95% confidence intervals were derived using Student’s t-test

^c^ Estimates and 95% confidence intervals for meta-analyses were derived using the inverse variance method

Supplementary Table 3. Differences in age at lung cancer diagnosis by tumor stage among females and males who never smoke

| Study/ Lung cancer histological subtype/tumor stage | Females | | Males | | Differences in age at diagnosis between females and males | |
| --- | --- | --- | --- | --- | --- | --- |
|  | n | Age at diagnosis, years, mean (SD) | n | Age at diagnosis, years, mean (SD) | Estimate (95% CI)^b^ | P-value |
| Taiwan Cancer Registry Population Study |  |  |  |  |  |  |
| Overall^a^ |  |  |  |  |  |  |
| Local | 5834 | 61.7 (11.4) | 1946 | 63.5 (12.4) | -1.8 (-2.4, -1.2) | 1.69x10^-8^ |
| Regional | 2236 | 64.6 (11.8) | 1020 | 67.7 (13.3) | -3.1 (-4.1, -2.2) | 2.18x10^-10^ |
| Distant | 13142 | 67.5 (13.0) | 5421 | 69.4 (13.0) | -1.9 (-2.3, -1.5) | 1.14x10^-20^ |
| LUAD |  |  |  |  |  |  |
| Local | 5449 | 61.6 (11.2) | 1687 | 62.7 (11.9) | -1.1 (-1.7, -0.5) | 7.81x10^-4^ |
| Regional | 1824 | 64.2 (11.3) | 592 | 65.3 (13.0) | -1.1 (-2.3, 0.1) | 0.07 |
| Distant | 11413 | 67.0 (12.8) | 3780 | 67.7 (13.1) | -0.6 (-1.2, -0.2) | 4.20x10^-3^ |
| SCC |  |  |  |  |  |  |
| Local | 81 | 66.1 (13.6) | 125 | 73.4 (10.9) | -7.3 (-10.9, -3.8) | 8.03x10^-5^ |
| Regional | 141 | 66.8 (13.2) | 263 | 72.0 (12.2) | -5.2 (-7.8, -2.6) | 1.35x10^-4^ |
| Distant | 549 | 67.5 (12.3) | 609 | 72.4 (11.5) | -4.8 (-6.3, -3.5) | 5.02x10^-12^ |
| Cancer Prevention Study-II Nutrition Cohort |  |  |  |  |  |  |
| Overall^a^ |  |  |  |  |  |  |
| Local | 69 | 75.7 (7.7) | 30 | 75.7 (7.5) | 0.01 (-3.3, 3.3) | 0.99 |
| Regional | 52 | 74.7 (7.3) | 23 | 77.5 (7.8) | -2.8 (-6.7, 1.1) | 0.15 |
| Distant | 99 | 74.7 (7.6) | 62 | 76.4 (7.7) | -1.7 (-4.2, 0.8) | 0.17 |
| LUAD |  |  |  |  |  |  |
| Local | 44 | 75.8 (6.4) | 24 | 75.6 (6.7) | 0.2 (-3.2, 3.6) | 0.91 |
| Regional | 35 | 75.7 (7.1) | 11 | 77.5 (6.8) | -1.7 (-6.8, 3.**2**) | 0.46 |
| Distant | 61 | 76.8 (7.1) | 37 | 75.6 (8.0) | 1.2 (-2.0, 4.4) | 0.46 |
| Cancer Prevention Study-3 |  |  |  |  |  |  |
| Overall^a^ |  |  |  |  |  |  |
| Local | 18 | 55.0 (8.6) | -- | -- | NA | |
| Regional | 5 | 61.0 (5.4) | -- | -- | NA | |
| Distant | 25 | 56.0 (6.7) | 5 | 56.0 (7.3) | -0.04 (-8.9, 8.9) | 0.99 |
| LUAD |  |  |  |  |  |  |
| Local | 10 | 56.8 (6.0) | -- | -- | NA | |
| Regional | 5 | 61.0 (5.4) | -- | -- | NA | |
| Distant | 20 | 55.5 (6.8) | -- | -- | NA | |

Abbreviations: SD, standard deviation; CI, confidence interval; LUAD, lung adenocarcinoma; SCC, squamous cell carcinoma; TCR, Taiwan Cancer Registry

^a^ overall lung cancer includes LUAD, SCC, as well as other histological subtypes

^b^ Estimated difference between mean age at diagnosis and 95% confidence intervals were derived using Student’s t-test
